# MicroRNA-Based Promotion of Human Neuronal Differentiation and Subtype Specification

**DOI:** 10.1371/journal.pone.0059011

**Published:** 2013-03-18

**Authors:** Laura Stappert, Lodovica Borghese, Beate Roese-Koerner, Sandra Weinhold, Philipp Koch, Stefanie Terstegge, Markus Uhrberg, Peter Wernet, Oliver Brüstle

**Affiliations:** 1 Institute of Reconstructive Neurobiology, LIFE & BRAIN Center, University of Bonn and Hertie Foundation, Bonn, Germany; 2 Institute for Transplantation Diagnostics and Cell Therapeutics, University Düsseldorf, Düsseldorf, Germany; University of Medicine and Dentistry of New Jersey, United States of America

## Abstract

MicroRNAs are key regulators of neural cell proliferation, differentiation and fate choice. Due to the limited access to human primary neural tissue, the role of microRNAs in human neuronal differentiation remains largely unknown. Here, we use a population of long-term self-renewing neuroepithelial-like stem cells (lt-NES cells) derived from human embryonic stem cells to study the expression and function of microRNAs at early stages of human neural stem cell differentiation and neuronal lineage decision. Based on microRNA expression profiling followed by gain- and loss-of-function analyses in lt-NES cells and their neuronal progeny, we demonstrate that miR-153, miR-324-5p/3p and miR-181a/a* contribute to the shift of lt-NES cells from self-renewal to neuronal differentiation. We further show that miR-125b and miR-181a specifically promote the generation of neurons of dopaminergic fate, whereas miR-181a* inhibits the development of this neurotransmitter subtype. Our data demonstrate that time-controlled modulation of specific microRNA activities not only regulates human neural stem cell self-renewal and differentiation but also contributes to the development of defined neuronal subtypes.

## Introduction

Based on their ability to self-renew and differentiate into any type of somatic cells, human embryonic and induced pluripotent stem (hES and iPS) cells represent an unrestricted source of specialized cells for basic and applied research. Different methods have been developed to derive neural stem cells and differentiated neural cell types from human pluripotent stem cells (hPSC) (reviewed in e.g. [1], [2]). We have established a protocol to obtain homogeneous long-term self-renewing neuroepithelial-like stem cells (lt-NES cells) from hPSC. Lt-NES cells can be continuously expanded in the presence of EGF/FGF2, and upon growth factor withdrawal differentiate into neurons and to a lesser extent also into glia [3]. Lt-NES cells have been successfully used to model human neurodegenerative diseases [4], [5] and represent a reductionist model for studying early stages of human neural stem cell differentiation *in vitro* [6].

MicroRNAs (miRNAs) are important modulators of self-renewal and differentiation [7]. During development miRNAs contribute to the establishment and maintenance of specific cell fates [8]. The efficacy of miRNA-based fate regulation has been demonstrated by the fact that overexpression of a few miRNAs suffices to reprogram somatic cells to iPS cells [9]–[11]. Moreover, miR-9/9* and miR-124 were shown to significantly contribute to the direct conversion of fibroblasts into neurons [12], [13].

During miRNA processing, the primary miRNA transcript is converted into a pre-miRNA that is subsequently cleaved by Dicer to yield a miRNA duplex. One strand (passenger-strand) is usually degraded whereas the guide-strand is incorporated into the RNA-induced-silencing complex (RISC). As part of RISC, miRNAs bind to motifs in the 3′UTR of mRNAs and induce translational repression or transcript degradation (reviewed in [14], [15]). MiRNAs can influence the expression of several transcripts, and their expression can be regulated through feedback loops with their targets. MiRNAs may integrate signals from different pathways, thereby affecting multiple events within a cell [16]. Therefore, miRNAs can be envisioned as tools to modulate differentiation and subspecification of neural stem cells and their progeny.

Here, we present the identification of novel miRNA functions in human neural stem cell differentiation. We show that miR-153, miR-324-5p/3p and miR-181a/a* contribute to shifting lt-NES cells from self-renewal to neuronal differentiation. We further demonstrate that miR-181a and brain-enriched miR-125b promote, while miR-181a* inhibits the generation of neurons of dopaminergic fate. Our work illustrates that the combination of miRNA expression analyses and time-controlled gain- and loss-of-function assays in lt-NES cells provides an efficient approach to identify novel miRNA functions affecting human neural stem cell fate.

## Materials and Methods

### Ethics statement

The human fetal midbrain RNA used in this study was extracted from human fetal midbrain samples (CS18-19, 6–7 weeks post conception) obtained from the MRC/Wellcome-Trust funded Human Developmental Biology Resource at Newcastle University (HBDR, http://www.hdbr.org), with appropriate maternal written consent and approval from the Newcastle and North Tyneside NHS Health Authority Joint Ethics Committee. HDBR is regulated by the UK Human Tissue Authority (HTA; www.hta.gov.uk) and operates in accordance with the relevant HTA Codes of Practice.

### Cell culture

Human embryonic stem cells (hES cells, I3 [17] and H9.2 [18] lines, kindly provided by Prof. J. Itskovitz-Eldor, Technion, Israel Institute of Technology, Haifa, Israel) were cultured on mouse embryonic fibroblasts (MEFs) according to standard protocols, or under feeder-free conditions on Matrigel (BD Biosciences) in MEF-conditioned medium. Human lt-NES cells (I3 and H9.2 lines), were maintained on polyornithin/laminin (Sigma-Aldrich) or Matrigel (BD Biosciences) with EGF and FGF2 (BD Biosciences) as previously described [3]. Default differentiation was performed on Matrigel in differentiation medium devoid of growth factors. To enrich differentiating lt-NES cultures with neurons of dopaminergic fate, cells were cultivated in DMEM/F12 supplemented with N2 (1∶100, Invitrogen, Life Technologies), 0.5 µM smoothened agonist SAG (Calbiochem), 100 ng/ml FGF8b, 20 ng/ml BDNF (both R&D Systems), and 0.2 mM ascorbic acid (Sigma-Aldrich) for 7 days. Lt-NES cells were cultivated for another 7 days in differentiation medium [3] supplemented with 20 ng/ml BDNF, 20 ng/ml GDNF, 2 ng/ml TGF-βIII (all from R&D Systems), 0.2 mM ascorbic acid and 0.5 mM dibutyryl-cAMP (both from Sigma-Aldrich).

### MicroRNA expression profiling

Small RNA fractions from two independent collections of, respectively, I3 hES cells, lt-NES cells self-renewing and differentiated for 15 (ND15) or 30 (ND30) days were prepared using the mirVana miRNA Isolation Kit (Ambion, Life Technologies). MicroRNA expression analyses were performed using a TaqMan microRNA multiplex qRT-PCR assay (Applied Biosystems, Life Technologies; [19]) according to the manufacturer's instructions. Briefly, aliquots of small RNA fractions corresponding to 5000 cells were subjected to reverse transcription employing 330 different miRNA-specific stem-loop primers followed by a multiplex pre-amplification PCR step. Subsequently, expression signals were determined by TaqMan qRT-PCR using the ABI Prism 7900 HT Sequence Detection System (Applied Biosystems, Life Technologies). MicroRNAs with a Ct value (threshold cycle) over 28 were considered as not significantly expressed. Raw Ct values were normalized to the median Ct value of all expressed miRNAs and further analyzed using the ΔΔCt method. Normalized miRNA expression in lt-NES cells, ND15 and ND30 neuronal differentiating cultures was compared to that in hES cells and analyzed by a two-step unsupervised clustering method using the Cluster 3.0 [20], [21] and the TreeView 1.1.3 [22] softwares.

### SYBR Green-based quantitative real-time RT-PCR (qRT-PCR) analyses

Total RNA samples were extracted using peqGOLD TriFast (Peqlab), DNaseI-treated (Invitrogen, Life Technologies) and quantified by Nanodrop (Thermo Scientific). For miRNA expression analysis, cDNA was synthesized using the miScript Reverse Transcription (RT) Kit (Qiagen). Quantitative real-time RT-PCR reactions were performed with miScript SYBR Green PCR Kit (Qiagen) on an Eppendorf Mastercycler. As forward primers, DNA oligonucleotides with sequence corresponding to the mature microRNAs were used. As reverse primer, the miScript Universal Primer provided by the miScript SYBR Green PCR Kit was used. PCR products were assessed by dissociation curve and gel electrophoresis. Data were normalized to RNU5A (fw: GTG GAG AGG AAC AAC TCT GAG TC) or miR-16 levels and analyzed using the ΔΔCt method. For mRNA expression analyses, cDNA was generated using the iScript cDNA Synthesis Kit (Bio-Rad). Quantitative real-time RT-PCR reactions were performed on an ABI Prism 7900 HT Sequence Detection System (Applied Biosystems, Life Technologies) using SYBR Green dection method. Primers used were: 18S fw: TTC CTT GGA CCG GCG CAA G, 18S rev: GCC GCA TCG CCG GTC GG; DAT fw: CAT CTA CGT CTT CAC GCT CCT, DAT rev: GTC ATC TGC TGG ATG TCG TC; GAD1 fw: CTT GTG AGT GCC TTC AAG GAG, GAD1 rev: TGC TCC TCA CCG TTC TTA GC; NURR1 fw: GGG CTG CAA AGG CTT CTT TA, NURR1 rev: ACA GCC AGG CAC TTC TGA AA; TH fw: ACT GGT TCA CGG TGG AGT TC, TH rev: TCT CAG GCT CCT CAG ACA GG. Data were normalized to 18S rRNA levels. PCR products were assessed by dissociation curve and gel electrophoresis. Normalized data were analyzed using the ΔΔCt method. For the qRT-PCR analysis of human brain samples, RNA extracted from whole human brain (18 fetal weeks, Agilent) and RNA extracted from human midbrain samples (CS18–19, 6–7 weeks post conception), provided by the Human Developmental Biology Resource [www.hdbr.org, Joint MRC (grant #G0700089)/Wellcome Trust (grant #GR082557)] were used.

### Non-radioactive Northern blotting

40 µg of each small RNA sample were separated on a 15% denaturing polyacrylamide gel and transferred onto a nylon membrane (Roche Applied Science). For size discrimination the Low Molecular Weight Marker 10–100 nt (USB, Affymetrix) was used. Digoxigenin (DIG)-labeled RNA oligonucleotide probes complementary to the mature miRNAs were synthesized by *in vitro* transcription in presence of DIG-11-UTP (Roche) using the mirVana Probe Construction Kit (Applied Biosystems, Life Technologies). U6 snRNA probes were used as loading control. After UV-crosslinking, the membrane was incubated with DIG-labeled RNA probes over night at room temperature. The membrane was then washed, and the hybridized probe was detected using the DIG Luminescent Detection Kit (Roche Applied Science) according to manufacturer's protocol. For re-probing, the membrane was stripped and hybridized again.

### Immunocytochemistry

For BrdU incorporation assays, lt-NES cells were incubated with bromodeoxyuridine (BrdU, 10 µM, Sigma-Aldrich) for 3.5 hours at 37°C, fixed in 4% PFA, permeabilized with 0.5% Triton X-100 and further processed and stained with an anti-BrdU antibody, as previously described [23]. To assess neuronal differentiation, PFA-fixed and permeabilized cells were stained with anti-β-III tubulin (Covance), anti-TH or anti-GAD65/67 (both Millipore) antibodies. Cells were counterstained with 4′,6-Diamidin-2-phenylindol (DAPI, Sigma-Aldrich). For quantification three representative pictures for each condition were analyzed and at least 1000 cells were counted. For quantification of neurite length the ImageJ plugin NeuronJ [24] was used.

### Cloning of miRNA overexpression constructs

For overexpression of miRNA constructs, the LVTHM vector [25] was used. This vector was modified to carry a puromycin resistance gene instead of GFP. The puromycin resistance gene was amplified from pLVX-Tight-Puro (Clontech) using the following fw: ACC TGC AGC CCA AGC TTA CCA T and rev: AGG TTG ATT GTT CCA GAC GCG C primers, and inserted in substitution of the GFP gene using PmeI and NdeI restriction sites. Next, the genomic loci (including the precursor and flanking sequences) of the miRNAs of interest were amplified from genomic DNA of lt-NES cells and cloned into the modified LVTHM vector using MluI and ClaI restriction sites. Primers used were: miR-124 fw: TGG TCC CTT CCT CCG GCG TT, miR-124 rev: ACA GGC TGC ACA CCT CCC CA; miR-125b fw: TAT ATG CGC CCC CAG ATA CT, miR-125b rev: CAT AGC AGC CAA CAC GCT AT; miR-153 fw: GCT GCC TGT TTC CTC T, miR-153 rev: AAT CCA GAG ATC CTC C; miR-181a/a* fw: TGT GAT GTG GAG GTT TGC, miR-181a/a* rev: AGT CCT GGT GTG TCC A; miR-324-5p/3p fw: GAG GTT GCA TAG TTG GGA CA, miR-324-5p/3p rev: CTG GGG CTT TCT TCC CAG T. As shRNA scrambled control the sequence from the pSilencer construct (Ambion, Life Technologies) was used.

### Lentiviral transduction of lt-NES cells

Production of lentiviral particles was performed in HEK293FT cells (Invitrogen, Life Technologies) by calcium phosphate transfection of cloned miRNA expression constructs and helper plasmids, as previously described [26]. Viral particles in the supernatant were concentrated by ultracentrifugation at 19.600 rpm at 4°C for 1.5 hours in a Sorvall Surespin 630 rotor (Thermo Scientific) and resuspended in HBSS (Gibco-Invitrogen). Lt-NES cells were exposed to virus and incubated over night at 37°C and 5% CO2 in growth medium supplemented with 5 µg/ml polybrene (Sigma-Aldrich). The next day medium was changed. 72 hours post-transduction lt-NES cells were treated with puromycin (5 µg/ml, PAA) for at least 2 days.

### Transfection with synthetic miRNA mimics and inhibitors

Lt-NES cells were plated on Matrigel (BD Biosciences) coated dishes and transfected with 10 nM of miScript miRNA Mimic or 100 nM of miScript miRNA Inhibitor (Qiagen) using Lipofectamin 2000 (Invitrogen, Life Technologies) four hours after plating. As control, cells were transfected with AllStars Negative Control siRNA or with miScript Inhibitor Negative Control (both from Qiagen). Four hours after transfection, medium was changed to the differentiation medium described before. Lt-NES cells were transfected every 48 hours until day 11 of *in vitro* differentiation, and replated after every third transfection. Cells were fixed and analyzed after 7 or 15 days.

### Statistical analysis

The data are presented as mean + SEM. Statistical significance, unless otherwise stated, was analyzed by two-tailed Student's t-test for control and experimental conditions, and p≤0.05 was considered to be statistically significant.

## Results

### Identification of microRNAs associated with human neuronal lineage

**Figure 1:**
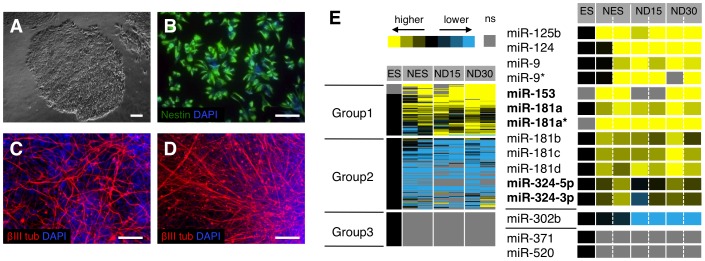
Analysis of microRNA expression in human ES cells, lt-NES cells and derived neural progeny. (**A**) Phase contrast image of a human ES cell colony (I3 line). (**B**–**D**) Immunofluorescent images of, respectively, self-renewing lt-NES cells stained for the neural precursor marker Nestin (**B**), and lt-NES cell cultures differentiated for 15 days (ND15; **C**), and 30 days (ND30; **D**), stained for the pan-neuronal marker β-III tubulin. DAPI labels nuclei. Scale bars = 100 µm. (**E**) Heat-map showing a hierarchical clustering of miRNA expression profiles in lt-NES cells (NES) and ND15 and ND30 differentiated neuronal cultures, compared to human ES cells (I3 ES, used as baseline). Relative expression levels in NES, ND15 and ND30 are displayed as log2 ratios compared to hES cells (yellow, expression increases; blue, expression decreases; “ns” stays for no significant expression). Representative miRNAs for the different expression groups identified are shown enlarged. In bold are indicated the newly identified neural-associated miRNAs further studied in this work. Abbreviations: DAPI, 4′,6-diamidino-2-phenylidole; ES, embryonic stem cells; lt-NES, long-term self-renewing neuroepithelial-like stem cells.

Several hundreds of miRNAs are expressed in the human brain [27], but knowledge of their functions in neuron lineage development is rather scarce. To identify miRNAs associated with early stages of human neuronal differentiation, we analyzed miRNA expression profiles of human embryonic stem cells (hES cells, I3 line, Figure 1A), hES cell-derived neuroepithelial-like stem cells (lt-NES cells, Figure 1B) and their differentiated neuronal progeny (Figure 1C, D). We particularly selected two different time points during the *in vitro* differentiation of lt-NES cells: 15 days (ND15, Figure 1C), when about 20% of the cells express the pan-neuronal marker β-III tubulin, and 30 days (ND30, Figure 1D), when the number of β-III tubulin-positive neurons reaches more than 50% (Figure S1). We examined miRNA expression in these samples using the ABI microRNA multiplex TaqMan assay covering 330 human miRNAs [19]. As shown in the hierarchical clustering in Figure 1E (left panel), we identified three major groups of miRNAs exhibiting distinct expression patterns: Group 1, miRNAs with increased expression in lt-NES cells and differentiating neuronal cultures; Group 2, miRNAs with decreased expression in lt-NES cells and differentiating neuronal cultures; Group 3, miRNAs showing expression only in hES cells.

**Figure 2:**
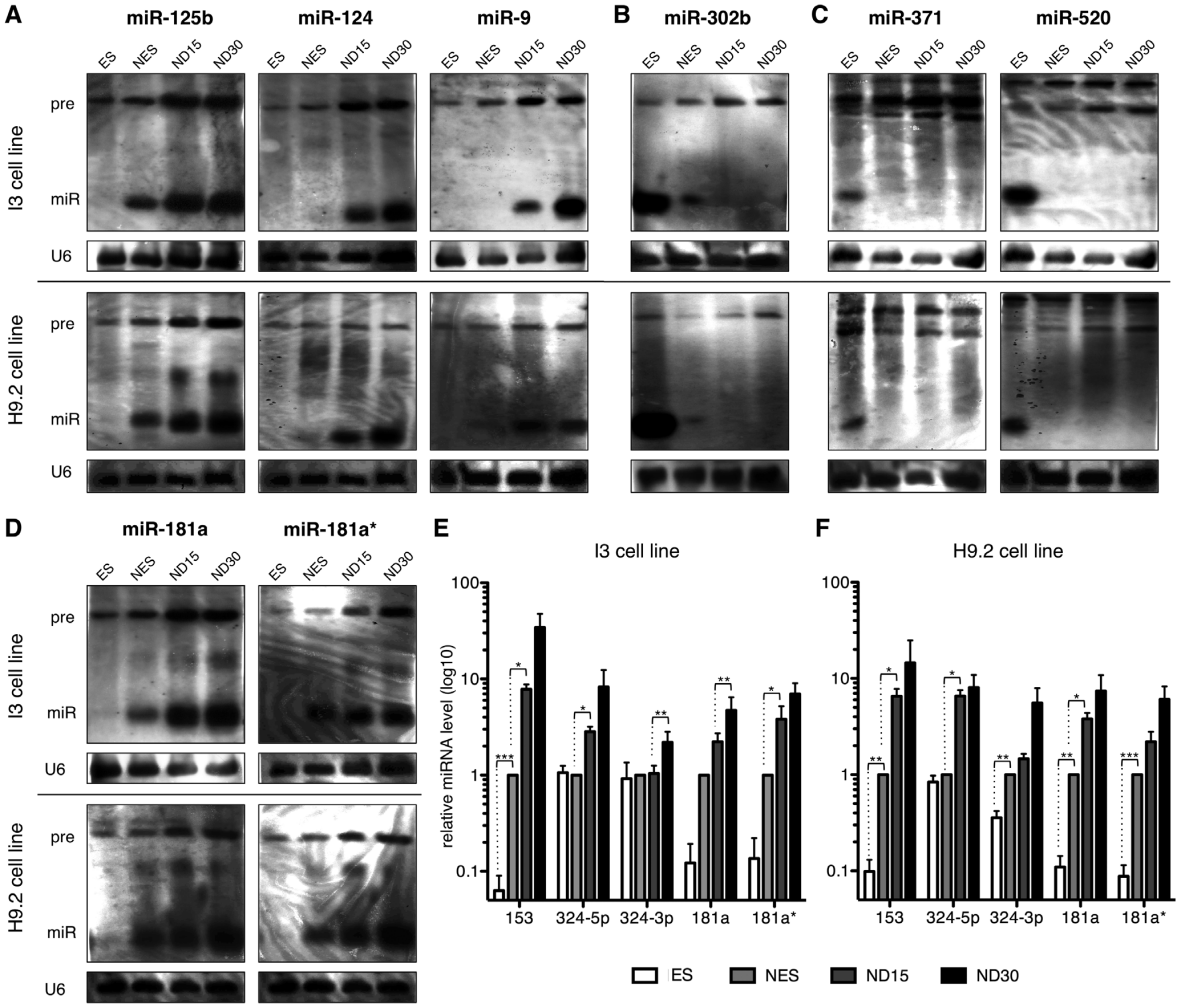
Validation of the identified microRNA expression patterns in two different cell lines. (**A**–**D**) Northern blot analyses showing expression of miRNAs in human ES cells (ES), lt-NES cells (NES) and lt-NES cells differentiated for 15 days (ND15) and 30 days (ND30) from the I3 and H9.2 cell lines. Representative miRNAs for the different expression groups identified are shown (Group 1, **A**; Group 2, **B**; Group 3, **C**). (**D**) Northern blot analyses showing expression of miR-181a and miR-181a* in the samples described above. Putative miRNA precursors are indicated by “pre”; mature miRNAs are indicated by “miR”. U6 snRNA was used as loading control. (**E, F**) qRT-PCR analyses monitoring expression of miR-153, miR-324-5p, miR-324-3p, miR-181a and miR-181a* in the samples described above from the I3 (**E**) and H9.2 (**F**) cell lines. Data are normalized to RNU5A snRNA reference levels and presented as average changes + SEM relative to expression in NES (baseline, set to 1; n = 3; *, p≤0.05; **, p≤0.005; ***, p≤0.0001). Abbreviations: ES, embryonic stem cells; lt-NES, long-term self-renewing neuroepithelial-like stem cells; qRT-PCR, quantitative real-time reverse transcription-polymerase chain reaction; snRNA, small nuclear RNA.

We validated the expression patterns of several miRNAs selected from each group in the I3 line by Northern blotting and extended this validation to a second cell line, i.e the H9.2 (Figure 2). Within Group 1, we found several brain-enriched and neuronal-associated miRNAs including miR-124, miR-125b and miR-9, which we have recently described in the context of lt-NES cell differentiation [28] All three miRNAs were up-regulated during neuronal differentiation of lt-NES cells (Figure 2A). Among the miRNAs expressed only in hES cells (Group 3), we found miR-371 and miR-520 (Figure 2C), which are known to be associated with pluripotency [29]–[31]. In contrast, miR-302b was found in Group 2 and showed expression both in hES cells and in lt-NES cells, but not in differentiated neuronal cultures (Figure 2B). This evidence is consistent with previous data showing that miR-302 is expressed in neural stem cells (e.g. [32]).

As shown in the Northern blots of Figure 2A–C, we were able to detect both mature miRNAs and their corresponding precursors (pre-miRNAs). Interestingly, although the precursors of the miRNAs analyzed were detected in all samples tested (hES cells, lt-NES cells and derived neuronal progeny), the mature miRNAs were differentially expressed. Mature miR-125b, miR-124 and miR-9 were found expressed in neural cells (lt-NES, ND15, ND30) only, whereas their precursors were also detected in hES cells (Figure 2A). Conversely, mature miR-371, miR-520 and miR-302b (Figure 2B, C) appeared to be expressed in the stem cell populations only (hES and lt-NES), while their corresponding precursors were present both in stem cells and neuronal differentiating cultures.

Besides the known neural-associated miR-124, miR-125b and miR-9/9*, our profiling revealed many other miRNAs up-regulated during human neural lineage entry and differentiation. Among these, we found miR-153, miR-324-5p/3p and the miR-181 family (Figure 1E right panel), for which evidence from other studies points to potential roles in the nervous system. MiR-153 was suggested as brain-specific miRNA [33] and found preferentially expressed in neurons [34]. It was shown to down-regulate alpha-synuclein and amyloid precursor protein levels [34], [35], which play important roles in the pathogenesis of Parkinson's and Alzheimer's disease, respectively. Expression of miR-324-5p was shown to increase with the conversion of murine cerebellar granule cell progenitors (GCPs) into mature granule cells, where this miRNA contributes to differentiation and growth arrest by antagonizing Hedgehog signaling [36]. The striking up-regulation of all the members of the miR-181 family upon lt-NES cell differentiation (Figure 1E right panel) points to potential roles of these miRNAs in human neuronal lineage. Expression of miR-181 family members can be detected in a variety of tissues, with the highest levels found in the brain [37]–[39]. The miR-181 family comprises six members, five of which (miR-181a, miR-181a*, miR-181b, miR-181c and miR-181d) were included in the multiplex qRT-PCR assay. We studied miR-181a and miR-181a* in more detail, since they were found to be involved in drug-induced synaptic plasticity [40]–[42] and in ageing-induced neuronal cell death in the rodent brain [43]. Furthermore, miR-181a as well as miR-153 and miR-324-5p were shown to act as tumor suppressors in human brain cancer cells (miR-181a, [44]; miR-324-5p, [36]; miR-153, [45], [46]).

We validated the expression patterns of miR-181a, miR-181a*, miR-153, miR-324-5p and miR-324-3p both in I3 and in H9.2 cell lines, using Northern blotting and SYBR Green-based qRT-PCR assays (Figure 2D–F). Expression of both miR-181a and its sister strand miR-181a* was relatively low in hES cells, but was up-regulated in lt-NES cells and further increased upon differentiation (Figure 2D–F). The other members of the miR-181 family showed comparable expression patterns (Figure S2). Interestingly, and in analogy to the more ubiquitous expression of the precursors observed for the neural- and pluripotency-associated miRNAs described, miR-181a and miR-181a* precursors were detected in all cell types analyzed, while mature miRNAs were present mainly in the neural cells (lt-NES and differentiated cells, Figure 2D). SYBR Green-based qRT-PCR analyses confirmed that expression levels of miR-153 and miR-324-5p/3p are up-regulated in neural cells compared to hES cells and further increased upon differentiation (Figure 2E, F). Taken together, qRT-PCR profiling and Northern blotting enabled us to associate a number of distinct miRNAs with defined stages of human neural stem cell differentiation.

### Ectopic expression of miR-153, miR-181a/a* and miR-324-5p/3p shifts lt-NES cells from self-renewal to neuronal differentiation and promotes neurite outgrowth

**Figure 3:**
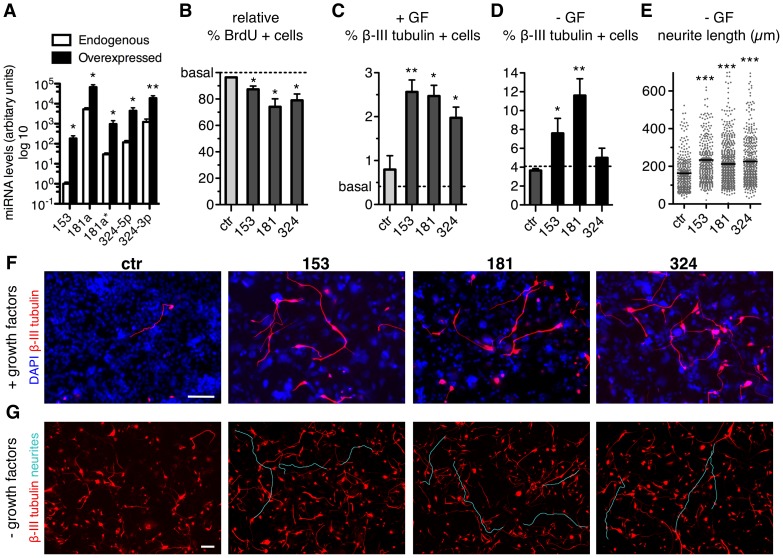
Overexpression of miR-153, miR-181a/a* or miR-324-5p/3p shifts lt-NES cells from self-renewal to neuronal differentiation. (A) Quantitative real-time RT-PCR analyses showing relative expression levels of mature miR-153, miR-181a, miR-181a*, miR-324-5p and miR-324-3p in lt-NES cells (I3 cell line) transduced with either LVTHM-ctr (ctr, for endogenous levels) or the respective LVTHM-miRNA overexpressing lentiviral constructs (LVTHM-miR-153, -miR-324-5p/3p, -miR-181a/a*). Data are normalized to miR-16 reference levels and presented as mean + SEM (n = 4; *, p≤0.05; **, p≤0.01; one-tailed Student's t-test). (B) Quantification of the relative percentage of BrdU-positive cells in lt-NES cell cultures transduced with the different specified LVTHM constructs, compared to untransduced cells (indicated as “basal”, with a BrdU incorporation rate imposed to be 100%). Data are presented as mean + SEM (n = 3; *, p≤0.05). (C, D) Quantification of the percentage of β-III tubulin-positive cells in LVTHM-transduced lt-NES cell cultures after 4 days in presence of growth factors “+ GF” (C), or after 7 days in differentiation medium devoid of growth factors “− GF” (D), compared to untransduced cells (basal, dashed line). Data are presented as mean + SEM (n = 4; *, p≤0.05; **, p≤0.01). (E) Scatter plot displaying length of single neurites (in µm) for each of the conditions described above after 7 days of differentiation. Data from three independent replicates are shown. Black lines indicate average neurite length (n = 3; ***, p≤0.0001). (F, G) Immunostainings for β-III tubulin in lt-NES cells transduced with the LVTHM constructs described above, and cultured for 4 days in presence of growth factors (F, DAPI labels nuclei) or 7 days in differentiation medium devoid of growth factors (G, neurites with more than 350 µm in length are labeled in cyan-blue). All scale bars  = 100 µm. Abbreviations: BrdU, bromodeoxyuridine; ctr, control; DAPI, 4′,6-diamidino-2-phenylidole; lt-NES, long-term self-renewing neuroepithelial-like stem cells; qRT-PCR, quantitative real-time reverse transcription-polymerase chain reaction.

As we identified an up-regulation of miR-153, miR-181a/a* and miR-324-5p/3p during the conversion of lt-NES cells into neurons, we became interested in whether an overexpression of these miRNAs in lt-NES cells would promote neuronal differentiation. We ectopically expressed in lt-NES cells the genomic locus of each selected miRNA using the LVTHM lentiviral vector [25], which was modified to express a puromycin resistance gene instead of the original GFP reporter. As control we used a scrambled non-targeting short-hairpin RNA (LVTHM-ctr). Upon transduction of lt-NES cells with LVTHM-miR-153, LVTHM-miR-181a/a* or LVTHM-miR-324-5p/3p we observed a stable increase of the respective endogenous miRNA levels (Figure 3A). The rate of BrdU incorporation in lt-NES cells under self-renewing culture conditions was significantly reduced by 9.41±2.53%, 22.77±6.03% and 17.73±4,76% upon overexpression of miR-153, miR-181a/a* and miR-324-5p/3p, respectively (Figure 3B and Figure S3). In addition to an impairment of BrdU incorporation, we observed an increase in neuronal differentiation upon constitutive expression of each of the three miRNAs. The rate of spontaneous differentiation into β-III tubulin-positive neurons for lt-NES cells under self-renewing culture conditions (i.e. plus growth factors, + GF) was 0.48±0.15% (basal, Figure 3C, F). A significant increase in β-III tubulin-positive cells was observed after transduction of lt-NES cells with LVTHM-miR-153 (5.06±1.93 fold), miR-181a/a* (4.99±2.09 fold) or miR-324-5p/3p (4.36±2.22 fold) compared to LVTHM-ctr transduced cells (Figure 3C, F). Upon growth factor withdrawal (- GF) lt-NES cells started to differentiate, and after 7 days of differentiation 4.09±0.53% cells were found positive for β-III tubulin (dashed line, Figure 3D). Ectopic expression of miR-153 or miR-181a/a* during differentiation further increased the fraction of β-III tubulin-positive neurons to 7.60±1.58% or 11.59±1.79%, respectively (Figure 3D). Surprisingly, overexpression of miR-324-5p/3p had no significant impact under differentiating conditions. We also observed significantly longer neurites in lt-NES-derived neurons overexpressing miR-153, miR-181a/a* or miR-324-5p/3p, compared to neurons in control cultures (Figure 3E, G). These results indicate a positive impact of all three miRNAs on neuronal differentiation of human neural stem cells. Intriguingly, miR-324-5p/3p seemed to increase the number of neurons generated during propagation of lt-NES cells under self-renewing conditions (+ GF), but this effect was lost when EGF and FGF2 were withdrawn from the cultures.

Taken together our data demonstrate that miR-153, miR-181a/a* and miR-324-5p/3p contribute to the transition of lt-NES cells from self-renewal to neuronal differentiation. In addition, all three miRNAs show a promoting effect on neurite outgrowth during lt-NES cell differentiation. It remains to be clarified whether the latter phenomenon is a consequence of the induced early onset of neuronal differentiation or of a more direct impact of these miRNAs on neurite development.

### Divergent impact of microRNAs on neuron subtype determination

**Figure 4:**
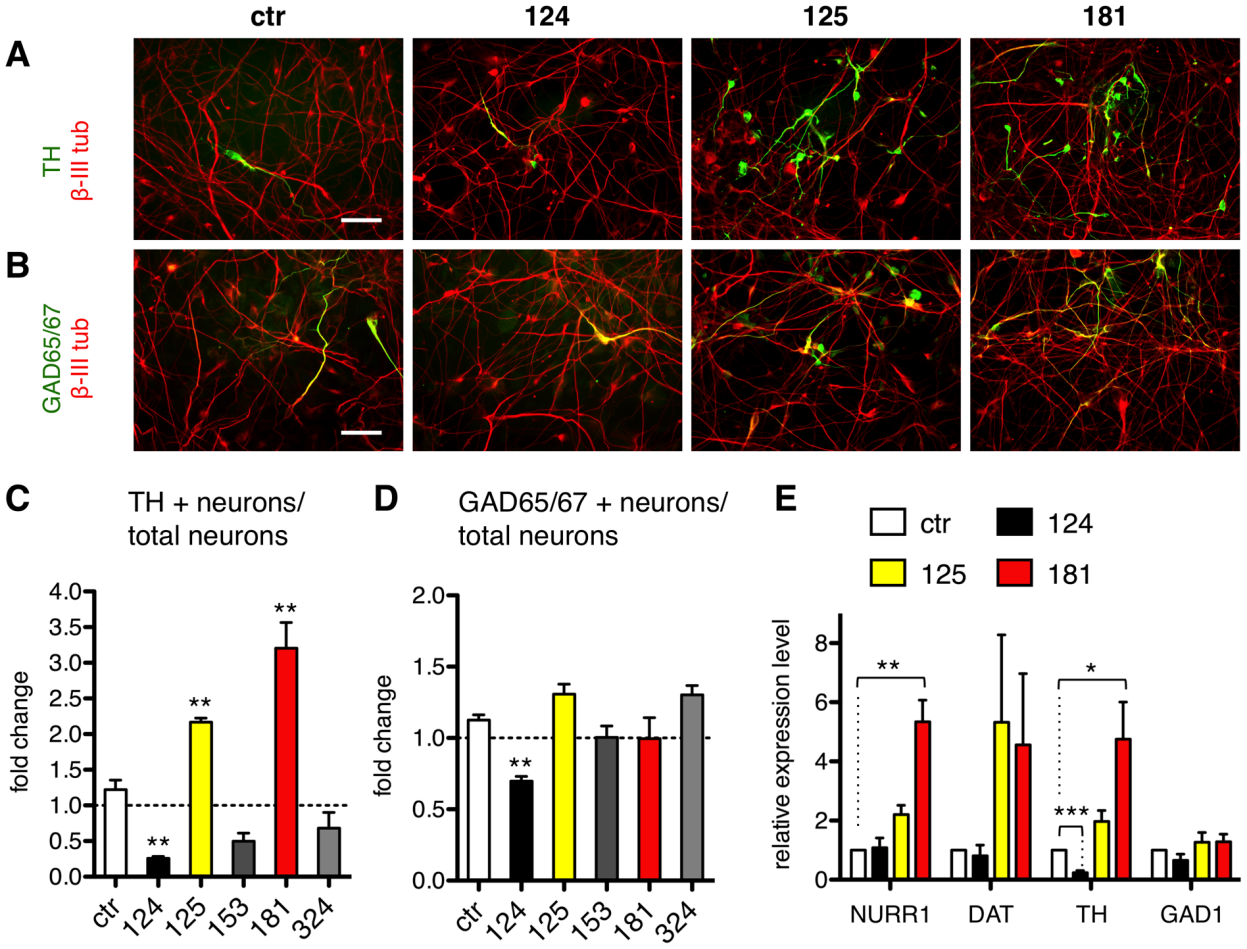
MiR-124, miR-125b and miR-181a/a*affect subspecification of lt-NES cell-derived neurons. (**A**, **B**) Immunostainings for β-III tubulin plus TH **(A)** or GAD65/67 (**B**) in lt-NES cells (I3 cell line) transduced with LVTHM-ctr or LVTHM-miR-124, -miR-125b and -miR-181a/a*, respectively, and differentiated for 15 days. Scale bars  = 100 µm. (**C**, **D**) Histograms showing the fold change in the number of TH-positive neurons (**C**) and GAD65/67-positive neurons (**D**) relative to the number of β-III tubulin-positive neurons in lt-NES cells transduced with LVTHM-ctr or LVTHM-miR-124, -miR-125b, -miR-153, -miR-181a/a* and -miR-324-5p/3p, compared to untransduced cells (equal to 1, dashed line). Data are presented as mean + SEM (n = 3; **, p≤0.01). (**E**) qRT-PCR analyses of NURR1, DAT, TH and GAD1 expression in 15 days differentiated lt-NES cell cultures overexpressing LVTHM-ctr, -miR-124, -miR-125b, and -miR-181a/a*, respectively. Data are normalized to 18S rRNA reference levels and presented as average changes + SEM relative to expression levels in LVTHM-ctr transduced lt-NES cells (equal to 1, n≥3; *, p≤0.05; **, p≤0.01; ***, p≤0.0001). Abbreviations: ctr, control; DAT, dopamine transporter; GAD, glutamic acid decarboxylase; lt-NES, long-term self-renewing neuroepithelial-like stem cells; NURR1, Nuclear receptor related 1 protein; qRT-PCR, quantitative real-time reverse transcription-polymerase chain reaction; rRNA, ribosomal RNA; TH, tyrosine-hydroxylase.

Judging from their transcription factor expression profile, lt-NES cells show an anterior-ventral hindbrain identity [3]. Upon induction of differentiation lt-NES cells give rise mainly to GABAergic interneurons, however they may be also patterned into different neuronal subtypes including e.g. dopamine neurons [3], [6]. Accumulating evidence supports a role for miRNAs in the development and maintenance of dopamine neurons. Mice lacking Dicer activity in postmitotic midbrain dopamine neurons show progressive loss of these cells and development of a Parkinson's disease-like phenotype [47]. The same study depicted miR-133b as a midbrain-enriched miRNA that affects maturation and function of midbrain dopamine neurons as part of a negative feedback loop with the midbrain dopaminergic determinant Pitx3. The actual impact of miR-133b on midbrain dopamine neuron development is unclear, since miR-133b null mice display normal dopamine neuron differentiation [48]. Very recently, miR-132 was shown to impair the differentiation of dopamine neurons from mouse ES cells by targeting the dopaminergic transcription factor Nurr1 [49]. We thus wondered whether the identified neural-associated miRNAs (miR-153, miR-181a/a*, miR-324-5p/3p) could also affect the development of specific neuronal subtypes with a particular focus on the dopaminergic lineage. We extended the analysis to the known neural-associated miR-124 and miR-125b for a comparison, since we have recently demonstrated their general contribution to neuronal differentiation of lt-NES cells [28]. We stained differentiated lt-NES cells for tyrosine-hydroxylase (TH), which is the rate-limiting enzyme in the synthesis of dopamine and a marker for dopaminergic as well as noradrenergic neurons. After 15 days of differentiation we observed that the number of TH-positive cells was significantly enhanced by ectopic expression of miR-181a/a* (2,72±0.48 fold) and miR-125b (1,81±0.20 fold) compared to control cells, even when normalized to the general increase of β-III tubulin-positive neurons (Figure 4A, C; Figure S4 shows absolute percentage). MiR-181a/a* and miR-125b had no significant influence on the number of GABAergic neurons, as shown by immunostainings for GAD65/67 and qRT-PCR analyses of GAD1 mRNA (Figure 4B, D, E). In contrast, ectopic expression of miR-124 led to a pronounced reduction in the numbers of both TH-positive neurons and GAD-positive neurons (Figure 4C, D). Overexpression of miR-153 and miR-324-5p, however, had no signifcant effect on either TH-positive or GAD-positive neurons (Figure 4C, D).

Quantitative real-time RT-PCR analyses revealed that transcript levels of NURR1 and DAT (dopamine transporter) were up-regulated in miR-125b or miR-181a/a* overexpressing lt-NES cultures compared to control cultures (Figure 4E), suggesting the dopaminergic nature of the TH-positive neurons identified.

Taken together these results indicate that the miRNAs under study influence neurotransmitter specification of differentiating human neural stem cells. More specifically, miR-181a/a* and miR-125b promote the yield of TH-positive neurons.

### Transient induction and inhibition of miRNAs modulate human neural stem cell fate

**Figure 5:**
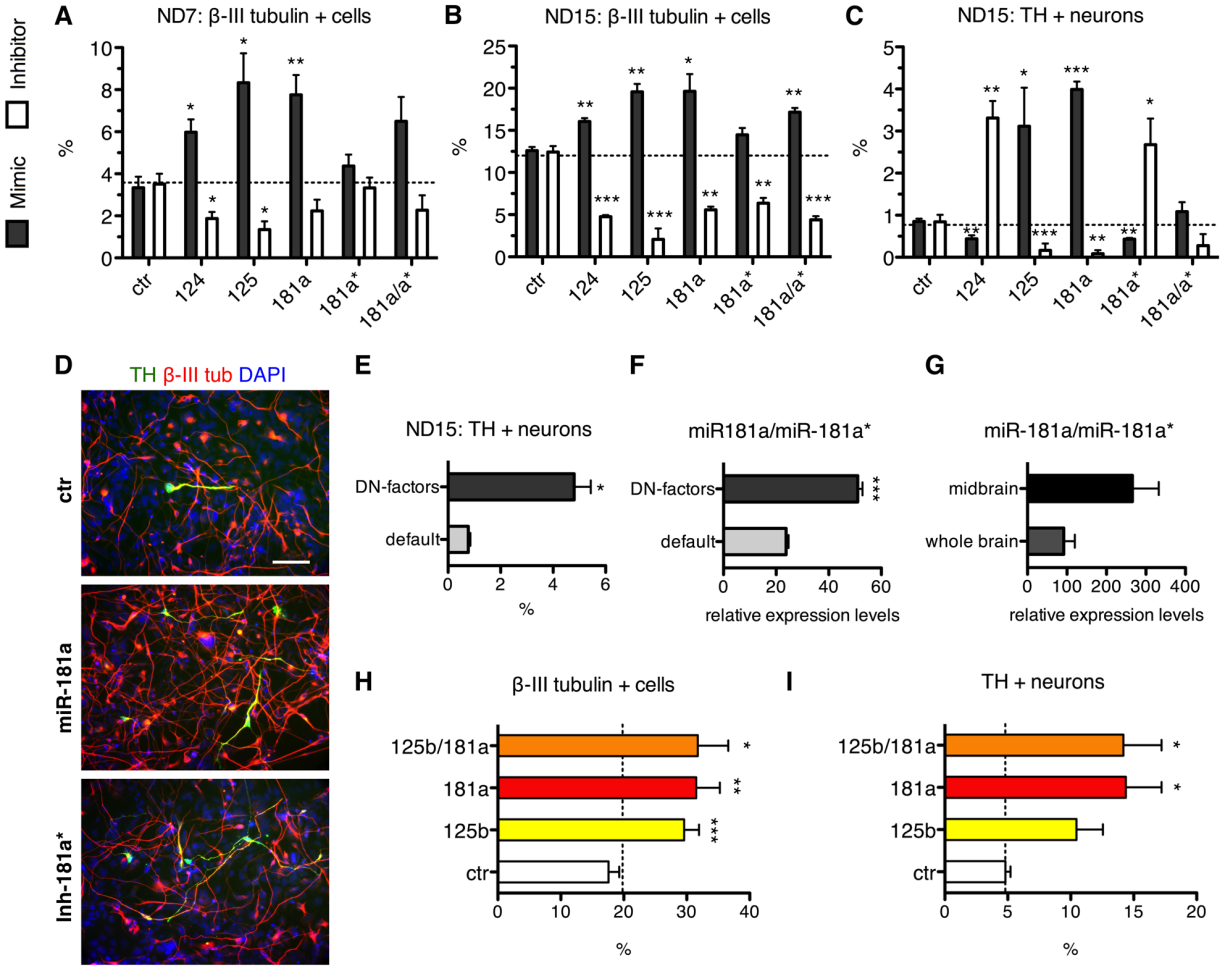
Transient modulation of microRNA activities impacts neuronal lineage development. (**A**, **B**) Histograms showing the percentage of β-III tubulin-positive cells in 7 days (**A**, ND7) and 15 days (**B**, ND15) differentiated lt-NES cells (I3 cell line) transfected with control (ctr), miR-124, miR-125b, miR-181a and miR-181a* mimics and inhibitors. (**C**) Histogram showing the percentage of TH-positive neurons in lt-NES cells transfected as described above and differentiated for 15 days. Data in **A**–**C** are presented as mean + SEM (n = 3; *, p≤0.05; **, p≤0.01; ***, p≤0.0001). (**D**) Representative immunostainings for β-III tubulin and TH in 15 days differentiated lt-NES cells transfected with ctr, miR-181a mimic or miR-181a* inhibitor. DAPI labels nuclei. Scale bar = 100 µm. (**E**) Histogram showing the percentage of TH-positive neurons in lt-NES cells differentiated for 15 days under default conditions (default) or in presence of factors that promote dopaminergic neuron differentiation (DN-factors). Data are presented as mean + SEM (n = 3; *, p≤0.05). (**F**) Histogram showing the ratio of miR-181a versus miR-181a* expression in lt-NES cells differentiated under default conditions or in presence of DN-factors, as assessed by qRT-PCR analysis. Data are normalized to RNU5A snRNA reference levels and are presented as mean + SEM (n = 3; ***, p≤0.0001). (**G**) Histogram showing the ratio of miR-181a versus miR-181a* expression in human fetal whole brain and in human fetal midbrain extracts, as assessed by qRT-PCR analysis. Data are normalized to miR-16 reference levels and presented as mean + SEM (n = 2). (**H**, **I**) Histograms showing the percentages of β-III tubulin-positive cells (**H**) and TH-positive (**I**) neurons in lt-NES cultures differentiated in presence of DN-inducing factors and transfected with miRNA mimics. The percentage of immunopositive cells in the mock-transfection controls in **A**–**C, H** and **I** is indicated by dashed lines. Abbreviations: ctr, control; DAPI, 4′,6-diamidino-2-phenylidole; DN, dopamine neuron, lt-NES, long-term self-renewing neuroepithelial-like stem cells; qRT-PCR, quantitative real-time reverse transcription-polymerase chain reaction; snRNA, small nuclear RNA; TH, tyrosine-hydroxylase.

In order to avoid genetic manipulation, miRNA activity may be modulated using synthetic miRNA mimics or inhibitors (Figure S5A, B). We tested whether transient increase or inhibition of the activity of the miRNAs under study suffices to affect lt-NES cell fate. Quantification of the percentage of β-III tubulin-positive cells in lt-NES cultures transfected with miRNA mimics and inhibitors confirmed that miR-124, miR-125b, miR-181a and miR-181a* are all impacting on neuronal differentiation (Figure 5A, B). Inhibition of these miRNAs resulted in a significant decrease in the number of neurons generated, which was already apparent 7 days after growth factor withdrawal and became particularly pronounced by 2 weeks of differentiation (Figure 5A, B).

Transfection of miR-125b mimic led to an increase in the percentage of TH-positive neurons (Figure 5C), which confirms the data obtained by lentiviral overexpression of the miR-125b locus. In line with the positive impact of miR-125b gain-of-function, inhibition of miR-125b in lt-NES cells impaired the generation of TH-positive neurons (Figure 5C). In contrast, inhibition of miR-124 raised the relative number of TH-positive neurons, despite the general reduction of neuronal differentiation observed in cultures transfected with miR-124 inhibitor (Figure 5B, C). Intriguingly, whereas miR-181a mimic transfection in differentiating lt-NES cells promoted the generation of TH-positive neurons (Figure 5C, D), transfection of miR-181a* mimic interfered with the differentiation of this neuronal subtype (Figure 5C). In line with this, transfection of miR-181a* inhibitor increased the number of TH-positive neurons (Figure 5C, D). Furthermore, co-transfection of lt-NES cell cultures with both miR-181a and miR-181a* mimics did not significantly alter the number of TH-positive neurons compared to control cultures (Figure 5C).

We next assessed whether all these effects are recapitulated in lt-NES cultures actively patterned towards a dopaminergic fate. To that end we treated lt-NES cells for one week with FGF8b and the smoothened agonist SAG to activate SHH signaling. Both morphogens are important for dopamine neuron specification during embryonic development [50]. Afterwards cells were differentiated for 7 days in presence of BDNF, GDNF, TGF-βIII, ascorbic acid and dibutyryl-cAMP. Use of these dopamine neuron-promoting factors (DN-factors) resulted in a 6.23±0.66 fold increase of TH-positive neurons after 15 days of *in vitro* differentiation, as compared to standard differentiation conditions (default, Figure 5E). Analysis of miRNA expression revealed that the ratio of miR-181a to miR-181a* is higher in lt-NES cultures differentiated according to the dopaminergic neuron-enriching protocol than in default differentiated cultures (Figure 5F). This suggests that the stability of the two miRNA species arising from the miR-181a/a* duplex may be cell fate-dependent. In further support of this hypothesis, the ratio of miR-181a to miR-181a* expression was found ∼ 2.5 fold higher in extracts from human fetal midbrain compared to commercial human fetal whole brain extracts - where tissue from forebrain is typically overrepresented (Figure 5G).

We next assessed whether co-transfection of miR-125b and miR-181a mimics could further increase the yield of TH-positive neurons in lt-NES cultures differentiated under dopamine neuron-enriching conditions (Figure 5H, I). Individual transfections of miR-125b or miR-181a mimics under these conditions resulted in a higher percentage of TH-positive neurons and increased in general the number of neurons compared to control cultures (Figure 5H, I). However, transfection of both mimics did not improve the yield of TH-positive neurons compared to the transfection of miR-181a alone (Figure 5I). This result suggests that both miRNAs might act on the same pathways. In conclusion, our work identifies a positive role of miR-125b and miR181a in the generation of neurons with a dopaminergic fate. Furthermore, it demonstrates that a transient increase or decrease in the functional levels of a few relevant miRNAs is sufficient to regulate the emergence of specific neurotransmitter subtypes in cultures of human neural stem cells.

## Discussion

In this work we sought to identify miRNAs associated with human neural stem cells and early stages of neuronal differentiation. We assessed miRNA signatures in human ES cells, ES-derived lt-NES cells and differentiated neuronal progeny and present a thorough functional analysis of newly identified neural-associated miRNAs. We show that miR-181a/a*, miR-153 and miR-324-5p/3p contribute to the shift from lt-NES cell self-renewal to neuronal differentiation. We provide evidence for a role of miR-125b and miR-181a in promoting the development of neurons with dopaminergic characteristics. We further demonstrate that miR-181a*, in contrast to its sister miRNA (miR-181a), interferes with dopaminergic differentiation. Importantly, our data indicate that transient modulation of specific miRNAs suffices to promote the generation of defined neuronal subtypes during lt-NES cell differentation.

### Cell type-specific microRNA processing

Northern blot analyses in hES cells and in their neural progeny (lt-NES cells and differentiated neurons) revealed differential expression of mature miRNAs, whereas the corresponding putative precursor forms were ubiquitously expressed. This indicates a cell type-specific processing of miRNA precursors. Similar observations were described e.g. for pre-let-7, which is processed into its mature form in neural stem cells but not in embryonic stem cells [51], [52] and pre-miR-138, which shows a differential processing pattern in different mouse brain tissues [53]. Context-specific precursor processing may represent a general principle in miRNA biogenesis, providing a time-saving mechanism for provision of specific active miRNAs [54]. As recently shown, the RNA binding protein FXRP1 (fragile X mental retardation syndrome-related protein 1) is involved in the regulation of miRNA biogenesis and participates in pre-miR-9 and pre-miR-124 processing in the mouse brain [55]. Our northern blot analyses of miR-124 and miR-9 expression patterns in hES cells and neural derivatives show that their precursor processing is compromised at the pluripotent stage. It will be interesting to explore whether FXRP1 has a role in the regulation of mature miR-124 and miR-9 expression during differentiation of human neural stem cells. Intriguingly, the maintenance of precursor expression in neuronal cultures for the pluripotency-associated miR-371 and miR-520, as well as for miR-302 might indicate that these miRNAs have further functional roles beyond the switch from self-renewal to differentiation.

### MicroRNAs controlling the switch from neural stem cell self-renewal to differentiation

We assessed miRNA expression profiles in lt-NES cells and in further differentiated neuronal cultures in order to identify novel miRNAs involved in early stages of neural stem cell differentiation. Among the miRNAs found up-regulated in lt-NES cells and differentiating neurons compared to hES cells, we selected miR-153, miR-324-5p/3p and miR-181a/a* for further analyses. Using gain-of-function strategies we demonstrated that these miRNAs contribute to the transition from lt-NES cell self-renewal to neuronal differentiation. Furthermore, loss-of-function experiments proved that miR-181a, as well as the neural-associated miR-124 and miR-125b are required for the differentiation of lt-NES cells into neurons.

MiR-153, miR-181a and miR-324-5p were found down-regulated in brain tumor cells and proposed to function as tumor suppressors [36], [44]–[46]. Specifically, reintroduction of miR-181a and miR-153 in glioblastoma cells impaired their proliferation [44], [45]. In analogy to these observations in a neoplastic background, we found that miR-153 and miR-181a/a* impair proliferation of non-tumorigenic human neural stem cells and promote their neuronal differentiation. In a similar analogy, miR-324-5p was found down-regulated in medulloblastoma cells and in murine self-renewing cerebellar granule cell progenitors, where it contributes to growth inhibition and differentiation by antagonizing the Hedgehog signaling [36]. These data correlate with our data in lt-NES cells, where miR-324-5p/3p inhibits proliferation while promoting, to a certain degree, neuronal differentiation. In order to investigate the molecular basis of these miRNA functions, *bona fide* targets associated with the observed phenotypes need to be identified. We recently showed that Notch signaling is critical for the regulation of lt-NES cell cycle progression [23]. Interestingly, miR-153, miR-181a/a* and miR-324-5p/3p are all predicted to target members of the Notch pathway. Among other putative targets are also genes directly related to cell cycle progression and self-renewal. In particular, miR-125b and miR-181a were shown to down-regulate Lin28 expression [52], [56], [57].

### MicroRNAs regulating the dopaminergic fate

Two studies have so far reported on specific miRNAs (i.e. miR-133b and miR-132) negatively affecting midbrain dopamine neuron development [47], [49]. To our knowledge, the work presented here is the first to describe miRNAs that rather promote the generation of neurons of dopaminergic fate. We demonstrate that overexpression of miR-181a and miR-125b during lt-NES cell differentiation induces an increase in the yield of TH-positive neurons and in the expression of dopaminergic markers, whereas inhibition of these miRNAs impairs the generation of this neurotransmitter subtype. Whether miR-181a or miR-125b have instructive roles in dopaminergic specification or rather act permissively during dopaminergic differentiation or both remains presently unclear. Addressing this question will require the identification of *bona fide* targets associated with dopaminergic lineage development. Target predictions combined to KEGG annotations show enrichment among the putative targets of miR-125b and miR-181a in neurotrophins and in members of signal cascades such as MAP kinase, WNT, Notch and TGF. Using HPRT-deficient human dopaminergic SH-SY5Y neuroblastoma cells as a model of Lesch-Nyhan disease, Guibinga et al. recently found that miR-181a down-regulates the expression of transcription factors relevant for dopaminergic development, i.e. EN1, EN2 and LMX1A [58]. While this finding stands in apparent contrast to our proposed dopaminergic fate-promoting effect of miR-181a, it could also be due to differences between the cellular model systems employed.

Intriguingly, the sister strands miR-181a and miR-181a* have opposite effects on the generation of TH-positive neurons. By modulating the activities of these two miRNAs individually using mimics and inhibitors we observed that miR-181a contributes to, while miR-181a* impairs the differentiation of TH-positive neurons. Interestingly, co-transfection of lt-NES cells with both miRNA mimics did not lead to any change in the yield of TH-positive neurons, suggesting that the two miRNAs neutralize each other. This result stands in contrast with data obtained from lentiviral overexpression of the miR-181a/a* locus and further indicates that modulation of individual miRNA activities is necessary to decipher the functions of each member of a miRNA duplex. Sequencing analyses [59], as well as our data, indicate that miR-181a is the dominant miRNA generated from the miR-181a/a* loci. This differential expression is maintained in lt-NES cells upon transduction with LVTHM-miR181a/a* (Figure S6). However, upon co-transfection of the respective mimic, miR-181a* reaches higher expression levels (Figure S5A), which might induce phenotypes others than those observed upon lentiviral overexpression and even mask/counteract miR-181a function. Therefore, we conclude that during differentiation of lt-NES cells towards the dopaminergic lineage miR-181a* may act as antagonist of miR-181a. Our qRT-PCR data further show that the ratio of miR-181a versus miR-181a* expression is consistently higher in lt-NES cultures differentiated in presence of dopaminergic-inducing factors compared to control cultures, as well as in human fetal midbrain compared to whole fetal brain. These data suggest that the two miRNA strands exhibit differential stability and incorporation into RISC depending on the cell fate. While strand selection is considered to depend on the thermodynamic properties of the miRNA duplex (reviewed in [60]), recent reports point to a cell type-specific expression of sister miRNAs [61]–[63]. In light of this, our observations suggest that miR-181a/a* strand selection might be regulated by cell type-dependent mechanisms.

## Conclusions

Our approach of combining miRNA expression profiling and gain- and loss-of-function studies in a stable population of human ES-derived neural stem cells led to the identification of novel miRNA functions in human neuronal differentiation. It further proved that transient modulation of specific miRNA activities is sufficient to promote the differentiation of neural stem cells towards defined neuronal subtypes. While this work was aimed at deciphering fundamental miRNA functions in neural stem cells and not at promoting the dopaminergic fate for downstream biomedical applications, it would be interesting to assess the roles of miR-181a and miR-125b in recently established cell culture paradigms specifically optimized for the specification of dopamine neurons (e.g. [64]–[68]).

## Supporting Information

Figure S1 **Assessment of the number of lt-NES cell-derived neurons.** Quantification of the percentage of β-III tubulin-positve cells in lt-NES cultures (I3 cell line) differentiated for 15 days (ND15) and 30 days (ND30). Data are presented as mean + SEM (n = 3; ***, p≤0.0001). Abbreviations: lt-NES, long-term self-renewing neuroepithelial-like stem cells.
(TIF)

Figure S2 **Expression of miR-181 family members in hES cells, lt-NES cells and derived differentiating cultures.** Northern blot analyses showing expression of mature miR-181b, miR-181c and miR-181d in human ES cells (ES), lt-NES cells (NES) and lt-NES cells differentiated for 15 days (ND15) and 30 days (ND30) from the I3 and H9.2 cell lines. U6 snRNA was used as loading control. Abbreviations: ES, embryonic stem cells; lt-NES, long-term self-renewing neuroepithelial-like stem cells; snRNA, small nuclear RNA.
(TIF)

Figure S3 Overexpression of miR-153, miR-181a/a* and miR-324-5p/3p impairs the rate of BrdU incorporation in lt-NES cells. Immunostainings for BrdU in lt-NES cultures (I3 cell line) transduced with LVTHM-ctr and LVTHM-miR-153, -miR-181a/a* and -miR-324-5p/3p constructs. DAPI labels nuclei. Scale bar  = 100 µm. Abbreviations: BrdU, bromodeoxyuridine; ctr, control; DAPI, 4′,6-diamidino-2-phenylidole; lt-NES, long-term self-renewing neuroepithelial-like stem cells.
(TIF)

Figure S4 **Impact of miRNA overexpression on the percentage of TH-positive neurons in differentiating lt-NES cell cultures.** Histogram showing the percentage of TH-positive neurons in untransduced lt-NES cells (I3 cell line, dashed line) and in lt-NES cells transduced with LVTHM-ctr, -miR-124, -miR-125, -miR-153, -miR-181a/a* and miR-324-5p/3p constructs, respectively, after 15 days of differentiation. Data are presented as mean + SEM (n = 3; *, p≤0.05; **, p≤0.01). Abbreviations: ctr, control; lt-NES, long-term self-renewing neuroepithelial-like stem cells; TH, tyrosine hydroxylase.
(TIF)

Figure S5 **Relative miRNA expression levels in lt-NES cells upon transfection with mimics and inhibitors.** (**A**, **B**) Quantitative real-time RT-PCR analyses showing relative expression levels of mature miR-124, miR-125b, miR-181a and miR-181a* in lt-NES cell cultures (I3 cell line) upon transfection with the respective miRNA mimics (10 nM, **A**) or inhibitors (100 nM, **B**) compared to control transfected lt-NES cells (ctr, equal to 1). Data are normalized to RNU5A reference levels and presented as mean + SEM (n = 3; *, p≤0.05; **, p≤0.01; ***, p≤0.0001). Abbreviations: ctr, control; lt-NES, long-term self-renewing neuroepithelial-like stem cells; qRT-PCR, quantitative real-time reverse transcription-polymerase chain reaction.
(TIF)

Figure S6 Expression levels of miR-181a and miR-181a* in lt-NES cells upon overexpression of the miR-181a/a* locus. Northern blot analyses showing expression of mature miR-181a and miR-181a* in lt-NES cells transduced with either LVTHM-ctr (ctr) or with LVTHM-miR-181a/a* (181a/a*). U6 snRNA was used as loading control. Abbreviations: ctr, control; lt-NES, long-term self-renewing neuroepithelial-like stem cell; snRNA, small nuclear RNA.
(TIF)
