# Correction: MicroRNA-Based Promotion of Human Neuronal Differentiation and Subtype Specification

**DOI:** 10.1371/annotation/b03fbc42-8f70-4873-9cce-854e48249a13

**Published:** 2013-09-12

**Authors:** Laura Stappert, Lodovica Borghese, Beate Roese-Koerner, Sandra Weinhold, Philipp Koch, Stefanie Terstegge, Markus Uhrberg, Peter Wernet, Oliver Brüstle

There was an error in the Competing Interests Statement. The second sentence of the Competing Interests Statement should read: "The lt-NES cell technology is proprietary (DE197 56 864.4 and all derivatives), and lt-NES® is a registered trademark of LIFE & BRAIN GmbH."

