# Supplementary figures and images for: MicroRNA-Based Promotion of Human Neuronal Differentiation and Subtype Specification
Source: PLoS One. 2013 Mar 18;8(3):e59011. doi: 10.1371/journal.pone.0059011 (PMC3601127; doi:10.1371/journal.pone.0059011)

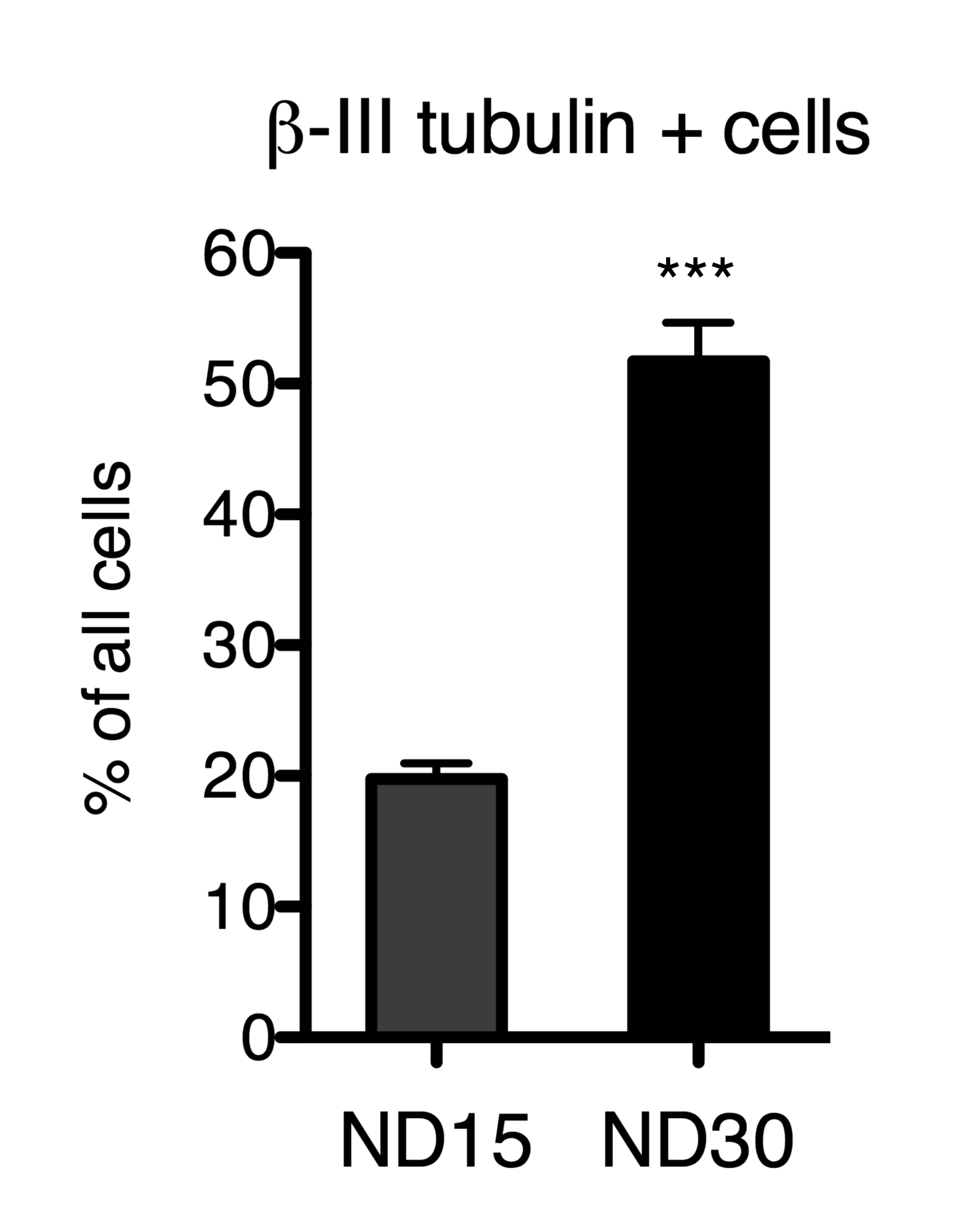

Supplement: Figure S1 — Assessment of the number of lt-NES cell-derived neurons. Quantification of the percentage of β-III tubulin-positve cells in lt-NES cultures (I3 cell line) differentiated for 15 days (ND15) and 30 days (ND30). Data are presented as mean + SEM (n = 3; ***, p≤0.0001). Abbreviations: lt-NES, long-term self-renewing neuroepithelial-like stem cells. (TIF) [file pone.0059011.s001.tif]

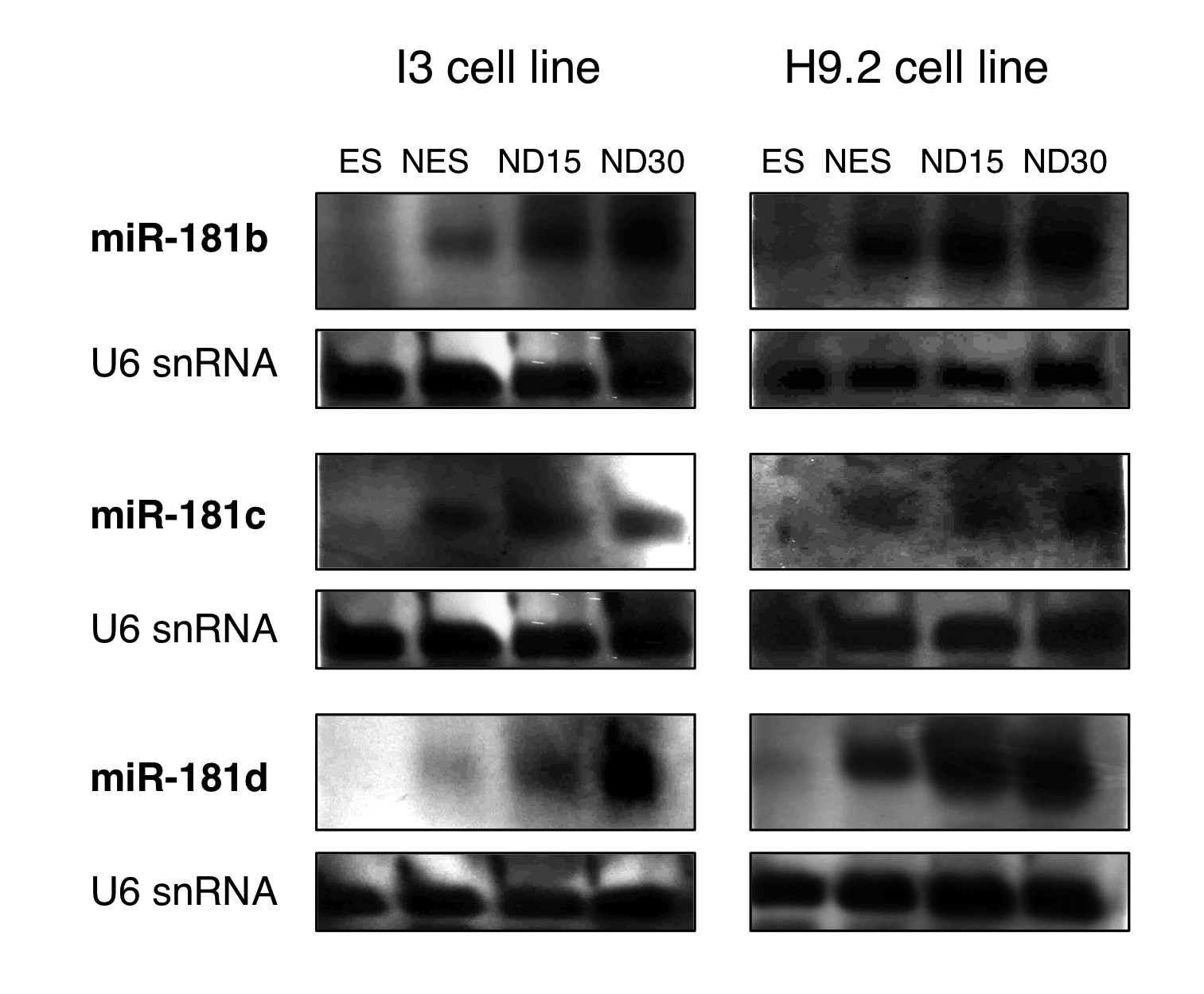

Supplement: Figure S2 — Expression of miR-181 family members in hES cells, lt-NES cells and derived differentiating cultures. Northern blot analyses showing expression of mature miR-181b, miR-181c and miR-181d in human ES cells (ES), lt-NES cells (NES) and lt-NES cells differentiated for 15 days (ND15) and 30 days (ND30) from the I3 and H9.2 cell lines. U6 snRNA was used as loading control. Abbreviations: ES, embryonic stem cells; lt-NES, long-term self-renewing neuroepithelial-like stem cells; snRNA, small nuclear RNA. (TIF) [file pone.0059011.s002.tif]

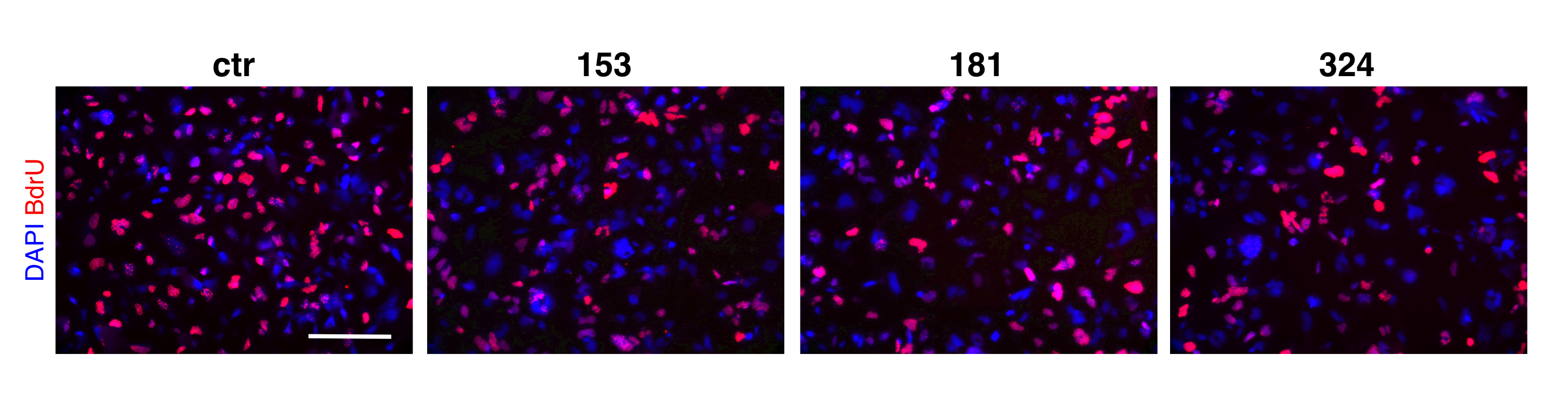

Supplement: Figure S3 — Overexpression of miR-153, miR-181a/a* and miR-324-5p/3p impairs the rate of BrdU incorporation in lt-NES cells. Immunostainings for BrdU in lt-NES cultures (I3 cell line) transduced with LVTHM-ctr and LVTHM-miR-153, -miR-181a/a* and -miR-324-5p/3p constructs. DAPI labels nuclei. Scale bar = 100 µm. Abbreviations: BrdU, bromodeoxyuridine; ctr, control; DAPI, 4′,6-diamidino-2-phenylidole; lt-NES, long-term self-renewing neuroepithelial-like stem cells. (TIF) [file pone.0059011.s003.tif]

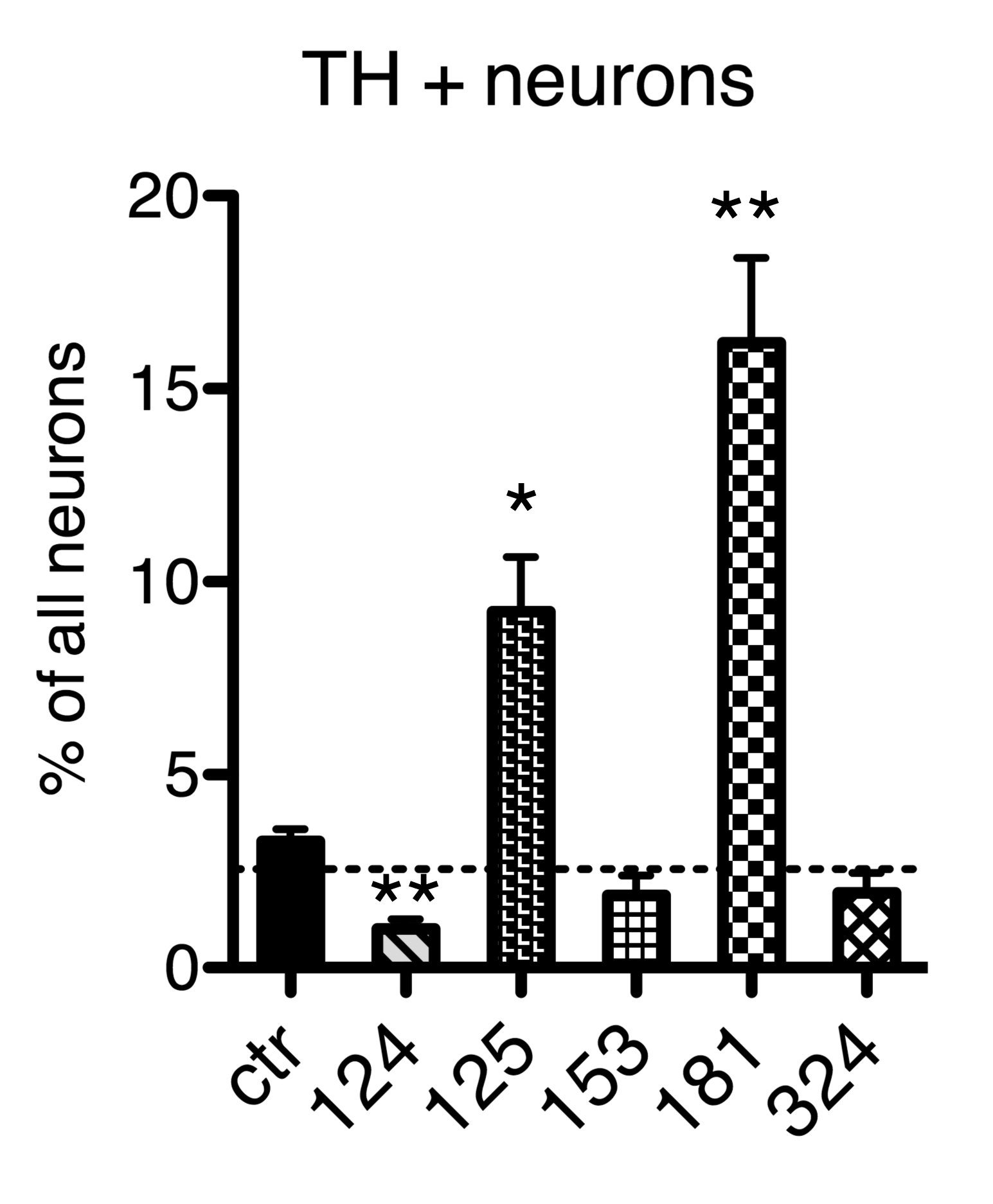

Supplement: Figure S4 — Impact of miRNA overexpression on the percentage of TH-positive neurons in differentiating lt-NES cell cultures. Histogram showing the percentage of TH-positive neurons in untransduced lt-NES cells (I3 cell line, dashed line) and in lt-NES cells transduced with LVTHM-ctr, -miR-124, -miR-125, -miR-153, -miR-181a/a* and miR-324-5p/3p constructs, respectively, after 15 days of differentiation. Data are presented as mean + SEM (n = 3; *, p≤0.05; **, p≤0.01). Abbreviations: ctr, control; lt-NES, long-term self-renewing neuroepithelial-like stem cells; TH, tyrosine hydroxylase. (TIF) [file pone.0059011.s004.tif]

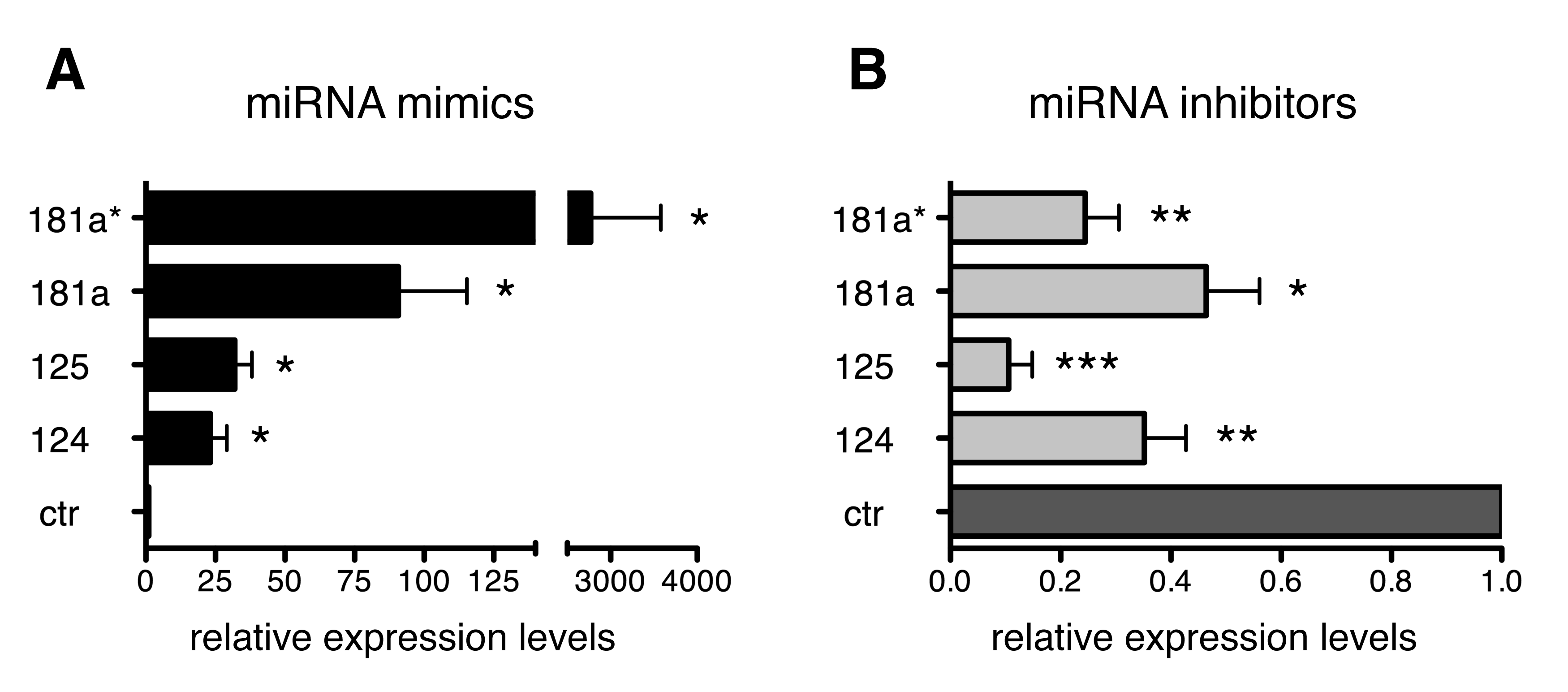

Supplement: Figure S5 — Relative miRNA expression levels in lt-NES cells upon transfection with mimics and inhibitors. (A, B) Quantitative real-time RT-PCR analyses showing relative expression levels of mature miR-124, miR-125b, miR-181a and miR-181a* in lt-NES cell cultures (I3 cell line) upon transfection with the respective miRNA mimics (10 nM, A) or inhibitors (100 nM, B) compared to control transfected lt-NES cells (ctr, equal to 1). Data are normalized to RNU5A reference levels and presented as mean + SEM (n = 3; *, p≤0.05; **, p≤0.01; ***, p≤0.0001). Abbreviations: ctr, control; lt-NES, long-term self-renewing neuroepithelial-like stem cells; qRT-PCR, quantitative real-time reverse transcription-polymerase chain reaction. (TIF) [file pone.0059011.s005.tif]

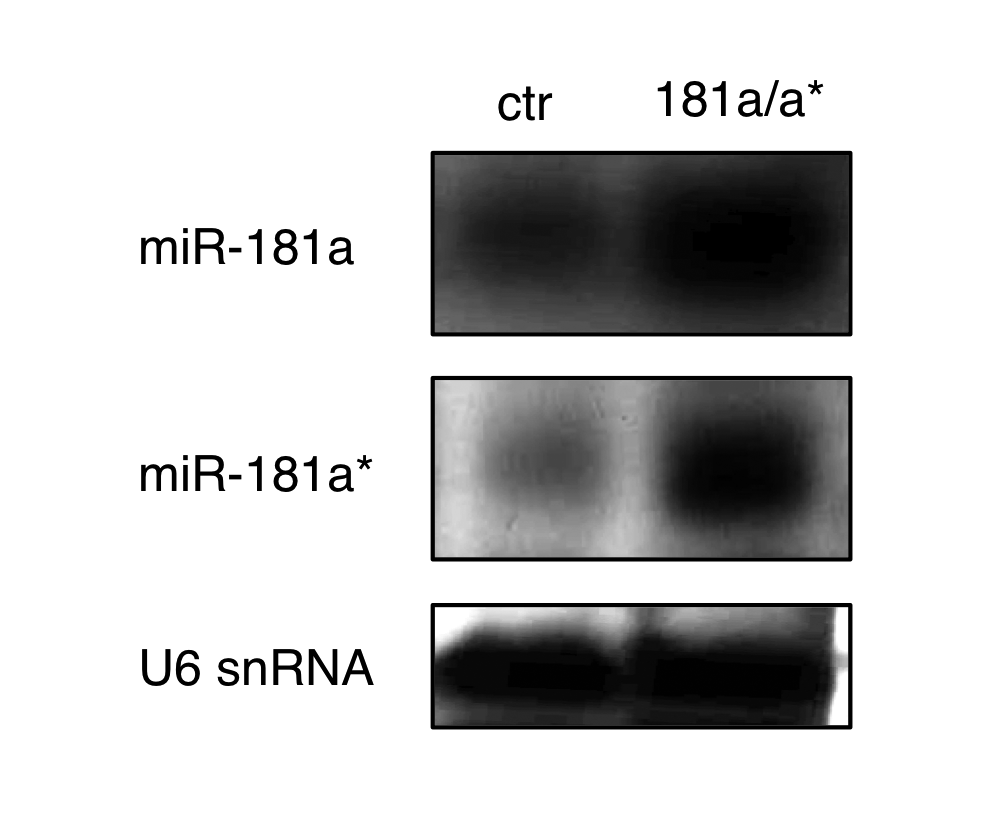

Supplement: Figure S6 — Expression levels of miR-181a and miR-181a* in lt-NES cells upon overexpression of the miR-181a/a* locus. Northern blot analyses showing expression of mature miR-181a and miR-181a* in lt-NES cells transduced with either LVTHM-ctr (ctr) or with LVTHM-miR-181a/a* (181a/a*). U6 snRNA was used as loading control. Abbreviations: ctr, control; lt-NES, long-term self-renewing neuroepithelial-like stem cell; snRNA, small nuclear RNA. (TIF) [file pone.0059011.s006.tif]
